# Analysis of Crack Cause of Parking Ratchet During the Manufacturing Process

**DOI:** 10.3390/ma18122821

**Published:** 2025-06-16

**Authors:** Haomin Fan, Xiaochun Xie, Jing Hu, Dandan Wang, Xulong An, Xiangkui Liu, Kunxia Wei, Wei Wei

**Affiliations:** 1 Jiangsu Key Laboratory of Materials Surface Science and Technology, Changzhou University, Changzhou 213164, China; 18161336497@163.com (H.F.); xxc81202@163.com (X.X.); ddwang@cczu.edu.cn (D.W.); axl@cczu.edu.cn (X.A.); xkliu@cczu.edu.cn (X.L.); kunxiawei@163.com (K.W.); benjamin.wwei@163.com (W.W.); 2 Huaide College, Changzhou University, Jingjiang 214500, China

**Keywords:** parking ratchet, carburizing and quenching, cracking, grinding, compressive stress, oxide inclusion

## Abstract

The parking ratchet is an important safety component of the car. Cracking occurs in the grinding surface during quenching and grinding processes after carburizing; thus, the goal of this research is to clarify the key cracking cause of the parking ratchet. Optical microscopy (OM), scan electronic microscope (SEM), energy dispersive spectroscopy (EDS), X-ray stress analyzer, etc., were used to systematically test and analyze the cracking cause. The results show that the microstructure of the parking ratchet with cracks after carburizing and quenching is normal, the residual stress of the surface is normal, with no oxide and decarburized layer within the crack areas, without burning during grinding, while it was found that oxide inclusions existed on the area of the crack, which is different from the normal specimens. Hence, a conclusion can be drawn that the cracking cause on the surface of the parking ratchet results from the oxide inclusions in the raw material. This study provides a feasible direction for the failure analysis and control of the cracks on parking ratchets during the manufacturing process.

## 1. Introduction

**Figure 1:**
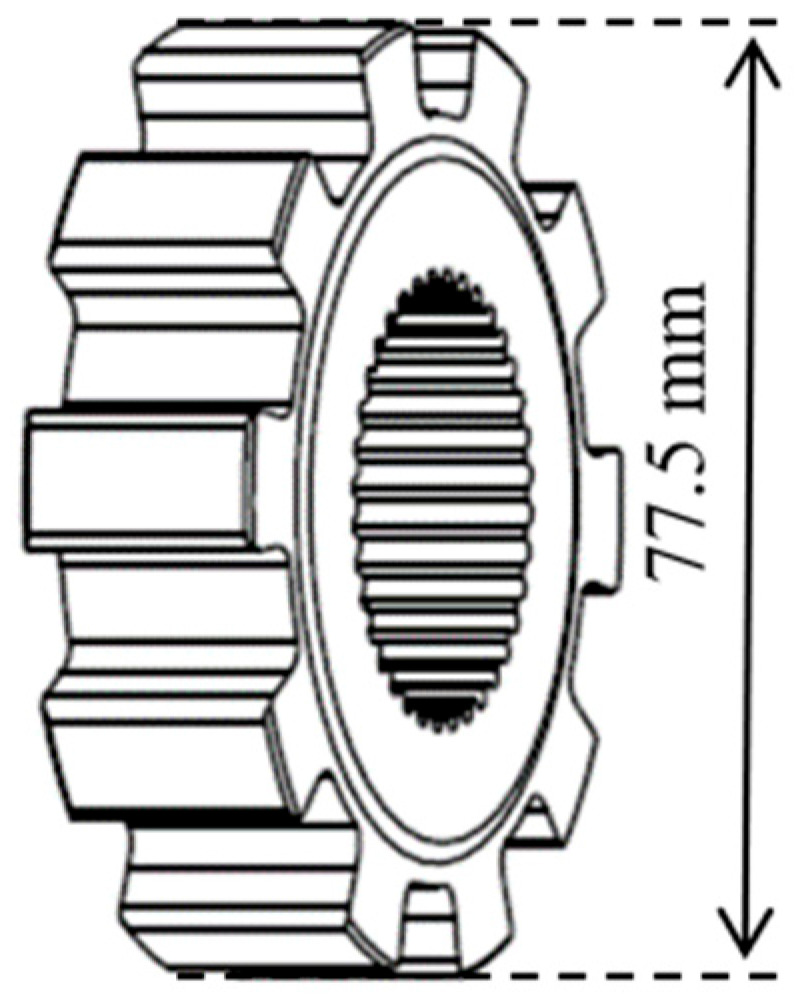
Parking ratchet structure.

The parking mechanism is a safety device that can achieve reliable parking in automotive automatic transmission, and it is an indispensable key mechanism in the transmission [1,2,3]. The parking ratchet is one of the key parts of the parking mechanism, and its typical structure is shown in Figure 1. During the service process, the parking ratchet is subjected to large alternating loads and long-time wear [4,5]. Therefore, its performance directly affects the quality and service life of the parking mechanism, and even affects the safety performance of the car.

**Figure 2:**
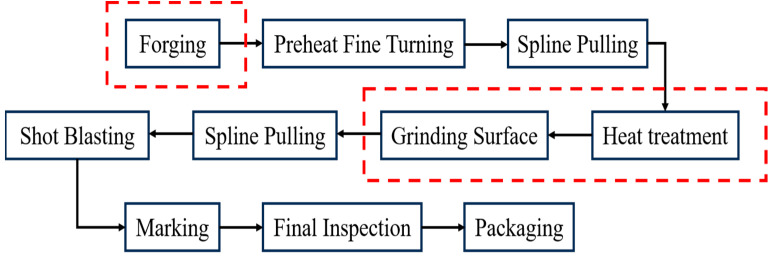
Manufacturing process of parking ratchets.

The main manufacturing process of the parking ratchet is illustrated in Figure 2, and the three steps that are prone to causing defects are marked by dotted red line boxes. Though the manufacturing process has been used for more than ten years, unfortunately, recently it was found by 100% magnetic particle inspection that cracks occurred on the parking ratchet after grinding, and with a proportion of 4.7%, which is called cracked parts here in this study. Therefore, it is of great significance to clarify the root crack cause in order to avoid this kind of failure during the manufacturing process and ensure the quality of the parking ratchet.

As it is known that determining the cause of crack formation and proposing control measures has always been a difficult task for the manufacturing industry, the researchers have carried out a lot of research on the root cause of cracking. Abbas Bahrami et al. analyzed the micro morphology of the C-Mn constructional steel heavy plates and the result of energy dispersive spectroscopy (EDS), and determined the root cause of cracking is V-shaped grooves on the surface of as-cast slabs [6]. Byoungchul Hwang et al. analyzed the microstructure of the cracked region, and demonstrated that the root cause of cracking is the iron oxide layer formed by inhomogeneous oxidization along the slanted ferrite–pearlite band structure in the side region within 30 mm from the plate edge was intruded into the interior after hot rolling [7]. Spyros A. Papaefthymiou analyzed macro and micro morphology of the alloyed S355 heavy plates and the result of energy dispersive X-ray spectroscopy (EDS) showed that the root cause of the cracking is the hot shortness issues that were caused by high-copper (having its origin mainly from scrap or from mold’s wear due to bad lubrication) amounts in the steel [8]. Manidipto Mukherjee and Tapan Kumar Pal investigated the effect of alloy segregation and delta (δ) ferrite contents on surface cracking of three standard (i.e., AISI 304L, AISI 310S, and AISI 321 [9]) and two low nickel (i.e., LNi-1 and LNi-0.3) austenitic stainless steels (ASS) during hot rolling, and found that the root cause of cracking is the segregation of Cu and Mn and excessive delta ferrite content [10].

To the best of our knowledge, no reports can be found on the causes of cracking in the parking ratchet, and no convenient methods can be used to effectively detect whether the cracks are caused by an improper heat treatment process. Therefore, the hardness, microstructure, and residual stresses after heat treatment, fracture morphology, and inclusions content in the raw materials were characterized, and the heat treatment limit test was carried out in this study. The goal is to clarify the root cause of cracking for the parking ratchets, so as to put forward the effective measures to control the formation of cracks.

Actual production was combined with the theory of heat treatment and material science in this study, which can provide ideas for the failure analysis of crack formation during heat treatment and machining of other products and provide a direction to check the rationality of the heat treatment process.

## 2. Material and Methods

The material of the parking ratchet is 20MnCrS5 HL, and the chemical composition is shown in Table 1, which meets the standard requirements.

**Figure 3:**
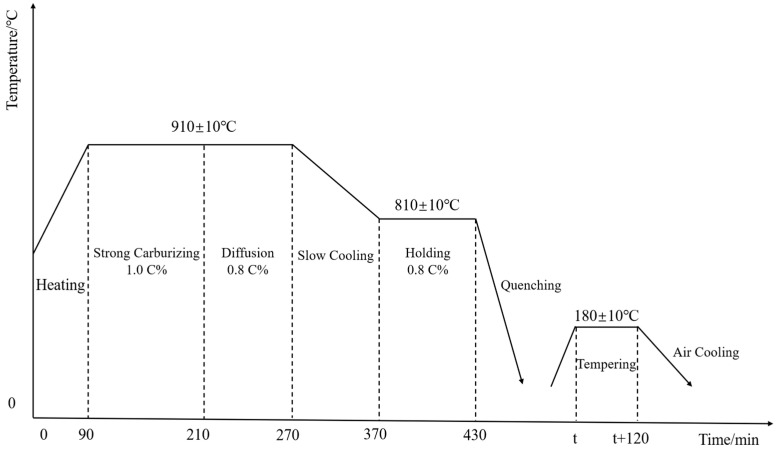
Heat treatment process of a parking ratchet.

Heat treatment is a key technology to improve the comprehensive service performance of automotive parking ratchets [11], and the heat treatment process of parking ratchets includes carburizing and quenching + low temperature tempering; the detailed heat treatment process is shown in Figure 3.

The technical requirements of the parking ratchet after heat treatment are as follows: the surface hardness is not less than 59 HRC; the depth of the carburized hardening layer (CHD) is 0.7–1.0 mm; the core hardness is 35–41 HRC.

After quenching and grinding the surface of the parking ratchet, a CJG-2000 fluorescent magnetic particle detector (Suzhou Industrial Park Kejing Instrument Co., Ltd., Suzhou, China) was used to carry out 100% magnetic particle inspection in order to pick out all the cracked parts. A ZHR4150LK Rockwell hardness tester (Time Group Inc., Beijing, China)was used to measure the surface hardness and core hardness (HRC) of the cracked parts, which was performed according to the standard ISO 6507-1:2018 [12], measuring three points and taking the average value. A Zwick/Roell ZHV30-S microhardness tester (ZwickRoell GmbH & Co. KG, Ulm, Germany) was used to measure CHD (CHD stands for Case Hardened Depth which means the vertical distance from the surface of the workpiece after heat treatment to the location of the 550 HV1), with a loading time of 5 s. Hardness values were tested at 0.1 mm intervals perpendicular to the surface inwards at the specified test position to a depth of 1.1 mm. The crack found by magnetic particle inspection was further observed by A ZEISS Vert.A1 metallographic microscope (Carl Zeiss AG, Oberkochen, Germany); metallographic grading was performed according to the standard QC/T 262-1999 [13].

An effective method of the heat treatment limit test was used to quickly and efficiently detect whether the cracks were generated during the quenching process or not, which can greatly improve the detecting reliability and efficiency compared with the traditional inspection method. The heat treatment limit test is conducted through the following process: preparing two groups of parking ratchet parts which are confirmed to be free of defects by magnetic particle inspection, each group has 5 pieces; adopting the following heat treatment process: the first group, carrying out normal carburizing and quenching process for two times continuously without tempering; the second group, after the normal carburizing and quenching process, carrying out tempering after 24 h; then, the two groups undergo magnetic particle detecting after 48 h to check whether there is any quenching crack or not; and then after completing the grinding surface, undergo magnetic particle detecting again to further check whether any cracks are formed during the grinding process or not.

A Proto-LXRD X-ray stress analyzer was used to detect the residual stresses of the cracked and normal parts after grinding the surface, and this detection was focused on the sub-surface layer, 500 μm, near the bottom of the inner spline groove, the test parameters were as follows: tube voltage 30 kV, tube current 20 mA, Cr target Kα radiation, V filter, collimated tube 1 mm in diameter, Fe(211) diffractive crystal plane, left and right 512 channel bit-sensitive detector, corresponding to the 2θ range of 20°, spatial ψ angle ± 45° within the optimized set up of 17 stations, isotropic diffraction geometry, Pearson VII peak fixing, material X-ray elasticity constant S2/2 = 5.92 × 10^−6^ MPa^−1^, and the test was conducted with the standards of ASTM-E915-2010, EN15305-2008 and GB7704-2017 [14,15,16]. An electrochemical corrosion test was carried out by Proto-8818 electrolytic polishing machine with 15 V, 2 A, and saturated NaCl hydrolysate. The depth of electrolytic polishing was measured by digital display micrometer.

Finally, the cracked area was completely sliced and an external force was applied to break the slice from the crack to form a fracture. The fracture morphology was observed by scanning electron microscope (SEM) and energy dispersive spectroscopy (EDS) was used to measure the elemental content in the fracture microregion.

## 3. Results and Analysis

### 3.1. Crack Inspection

**Figure 4:**
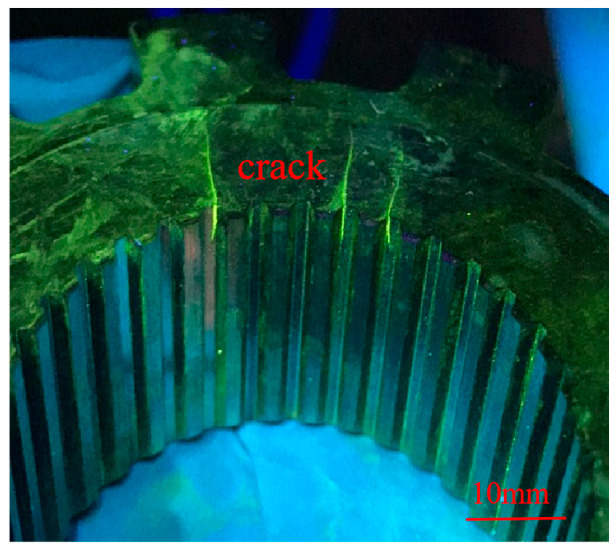
Crack revealed by magnetic particle inspection.

A CJG-2000 fluorescent magnetic particle detector was used to detect the cracks, considering that the generation of grinding cracks has a delayed nature, so after the completion of grinding, a waiting period of 48 h was incorporated to release the residual tensile stress prior to carrying out magnetic particle inspection on the workpiece in order to effectively avoid the presence of undetected defects. Once reaching the duration of 48 h, the parking ratchets were subjected to 100% magnetic particle inspection, and it was found that 4.7% parking ratchets had the same surface cracks, as shown in Figure 4.

**Figure 5:**
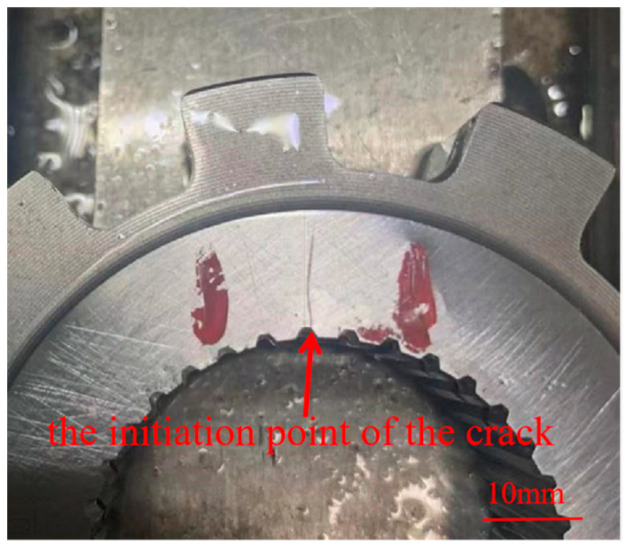
An overhead view of the cracks on the grinding surface.

In order to have a complete morphology of the crack, an overhead view was observed as shown in Figure 5. It can be seen that the crack is almost linear, the starting angle is located in the grinding surface and the inner spline boundary, and it extends from the inner spline groove to the vicinity of the plane edge; generally, cracks preferentially appear in the corner and edge position of the workpieces [17,18]. Meanwhile, it can be seen that there exists no grinding burn on the surface of the cracked area, so it can determine that the grinding process is reasonable, and the root cause of cracking for the parking ratchets is not due to the grinding process.

### 3.2. Analysis of Hardness and Depth of Carburized Hardened Layer

**Figure 6:**
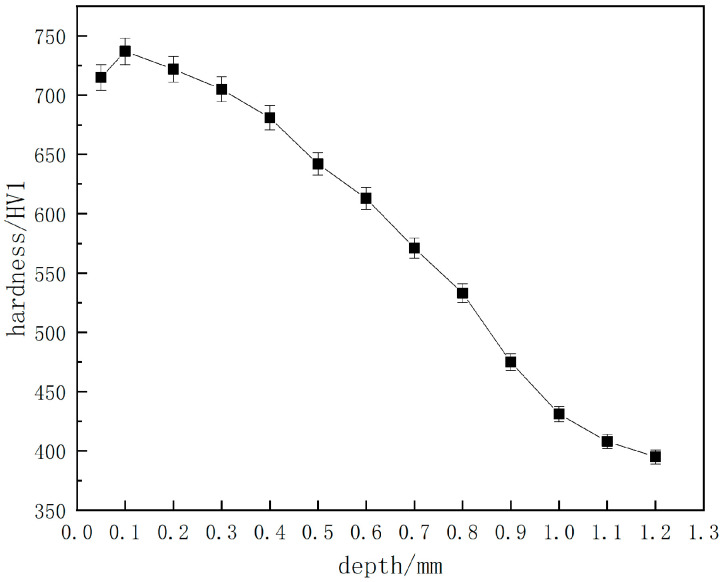
Cross-section hardness curve of a cracked parking ratchet.

A cracked parking ratchet is arbitrarily used for hardness and CHD tests, and the cross-section hardness curve is shown in Figure 6. The values of surface hardness, core hardness, and CHD are shown in Table 2. It can be seen that the average value of the surface hardness is 706.2 HV1, the core hardness is 393 HV1, and the CHD is 0.76 mm; all the values meet the technical requirements of the product.

**Figure 7:**
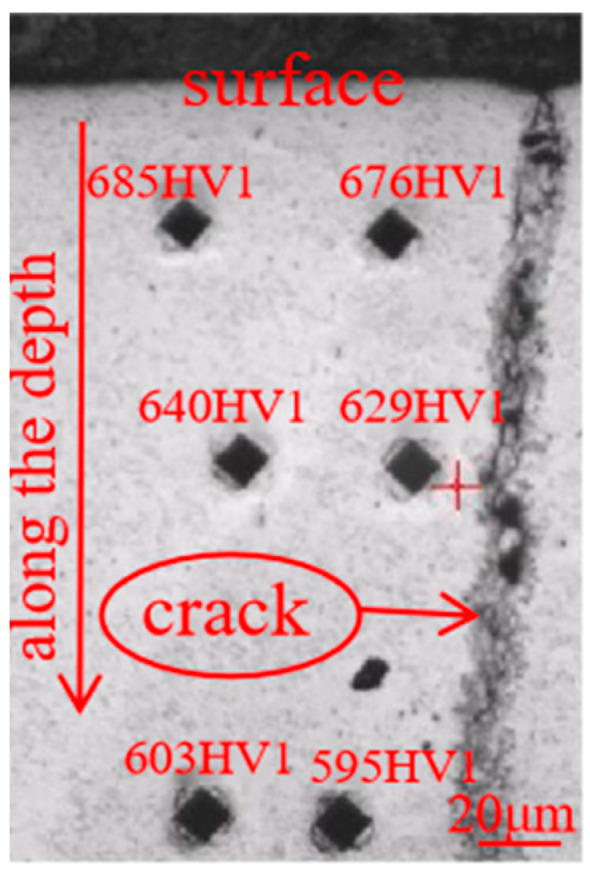
Microhardness near and away from the cracks.

Figure 7 presents the microhardness along the depth with different distance from the crack. It shows the hardness near the crack is 676 HV1, 629 HV1, and 595 HV1, while the corresponding parallel position away from the crack is 685 HV1, 640 HV1, and 603 HV1, respectively. It can be seen that the hardness decreases along the depth and is very close at the same depth near and away from the crack, though the hardness near the crack is a little lower than that away from the crack, which is because more internal stress can be released near the crack, leading to a lower stress hardening effect, and thus corresponding to a little lower hardness [19]. And because the microhardness test was conducted on the same base metal, it can be considered that the chemical composition keeps unchanged. And since the tested hardness at the same depth with different distance from the crack is very close, it implies that the microstructure at the same depth near and away from the crack is supposed to be almost the same, due to the well-known knowledge that the property of hardness is dependent on the chemical composition and microstructure.

### 3.3. Microstructure Observation

**Figure 8:**
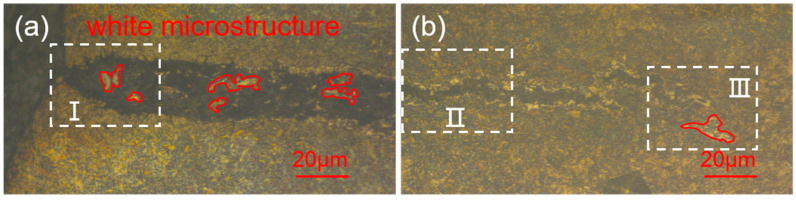
(**a**,**b**) Metallographic microstructure of the crack.

The microstructure of the cracked area under a 200× optical microscope is shown in Figure 8a,b. It can be seen that there is no typical characteristic of a decarburized layer [20] at the edge area of the crack, but white microstructure appeared at a certain area on the cracked surface as marked by a red circle, which is obviously abnormal microstructure.

**Figure 9:**
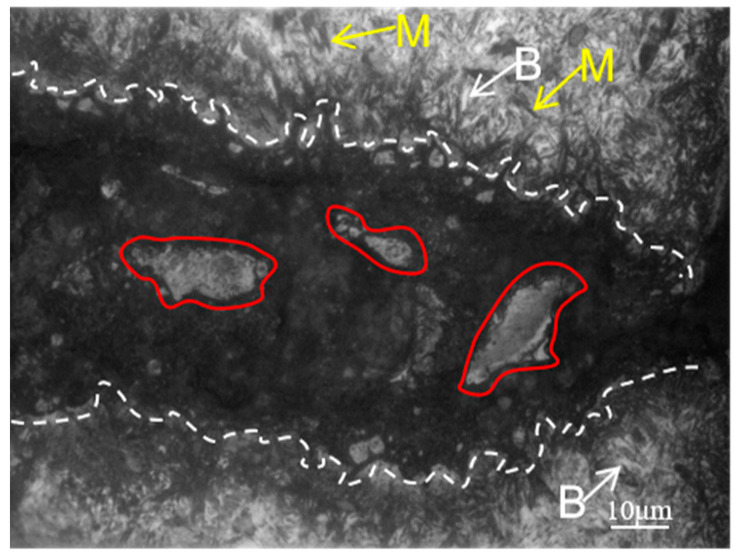
Metallographic microstructure of the cracked start area.

**Figure 10:**
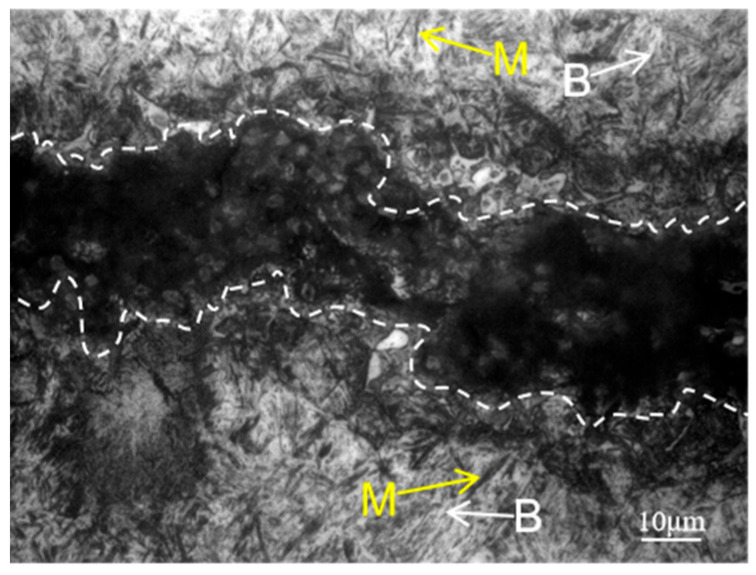
Metallographic microstructure of the cracked middle area.

**Figure 11:**
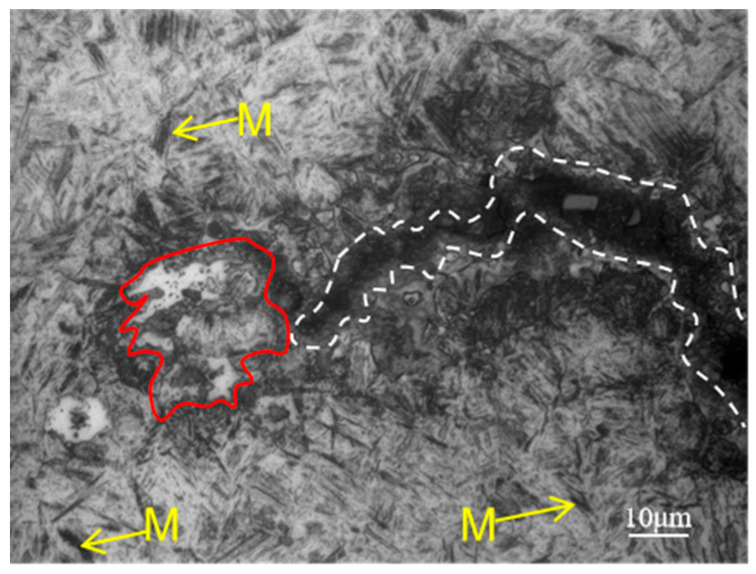
Metallographic microstructure of the cracked end area.

Higher magnification optical microscope images are presented in Figure 9, Figure 10 and Figure 11. The crack area is marked by a white dotted line and the microstructure of the crack start area is shown in Figure 9, which corresponds to zone I in Figure 8a. Since the start of the crack area occurred at the carburized area, a large amount of martensite marked by yellow arrows and a small amount of bainite marked by white arrows can be seen at the substrate near the crack. And it can be clearly seen that the white microstructure is totally different from the base microstructure of martensite and bainite in this area.

Figure 10, which corresponds to zone II in Figure 8b, shows the crack gradually narrows and is surrounded by white microstructure on both sides, as well as martensite and bainite around it, which can be inferred that the main crack may also split into a number of smaller cracks to form secondary cracks in the process of crack extension, due to the role of various obstacles, and hard martensite and grain boundaries having a blocking effect on the extension of the crack. From the secondary cracks near the white microstructure in the middle of the crack, this white microstructure is peeled off from both sides due to crack extension, which is why there exists no white microstructure in the cracked middle area, though there are white microstructures at the cracked start and end area.

Figure 11, which corresponds to zone III in Figure 8b, shows the microstructure of the end area of the crack. There is a white microstructure appearing on the matrix not far from the end of the crack, in other words, the crack marked by white has not yet extended to this area, thus proving that the formation of white microstructure is not due to the crack, and the cracks spread along the direction of the distribution of the white microstructure. At the same time, because the crack extends to the core area, a large amount of lath martensite marked by yellow arrows can be seen.

From Figure 8, Figure 9, Figure 10 and Figure 11, it can be seen that the white microstructures have no distinguishable morphology and exist before the appearance of crack, while the white microstructures may promote the formation of crack during heat treatment and grinding. Therefore, it can be determined that the white microstructure is supposed to be included in the raw material, and it has a strong tendency to result in cracking during the manufacturing of parking ratchets.

### 3.4. Heat Treatment Limit Test

The results of heat treatment limit test for the two groups are shown in Table 3. It can be seen there are no cracks found after 100% magnetic particle inspection after the heat treatment process and grinding surface process for the two groups of parking ratchets, which indicates that as long as no defects existed on the surface of the raw material [21,22], the internal stresses generated by the heat treatment process will not lead to quenching cracks, as well as the superposition of the grinding force will not cause the crack. In other words, the heat treatment process is confirmed to be reasonable by the heat treatment limit test.

### 3.5. Residual Stress Detection

**Figure 12:**
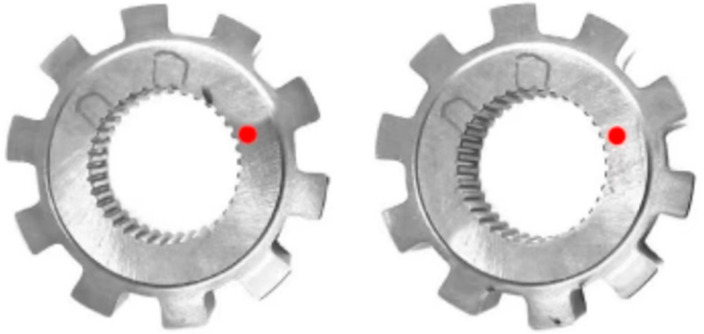
Schematic diagram of detection position of surface residual stress. Normal part (**left**); cracked part (**right**) (5×).

Figure 12 shows the schematic diagrams of the residual stress detection positions on the surfaces of the normal parts and the cracked parts after grinding, with the detection position which was marked by red dot close to the start position of the crack; the residual stress detection results are shown in Table 4, where the negative stress represents the residual compressive stress. It can be seen that although the surface and subsurface compressive stresses of the cracked parts are a little lower than those of the normal parts, while both of them belong to the expected compressive stress state; thus, it can be determined that they will not lead to crack formation, on the contrary, they can prevent the parts from forming surface cracks during the service due to the compressive stress state [23].

### 3.6. Morphology of the Crack Fracture

**Figure 13:**
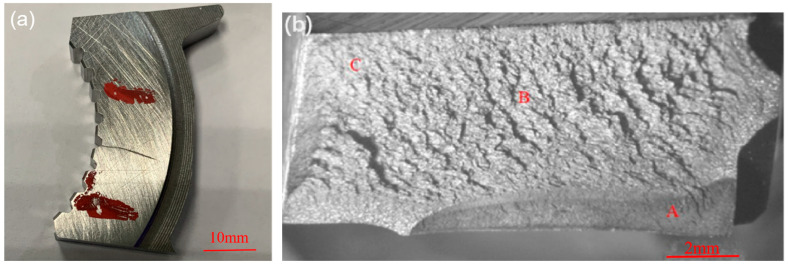
(**a**) Cracked part, (**b**) macroscopic appearance of the crack fracture.

In order to find out the generation source and propagation form of the crack, the cracked part was cut down as shown in Figure 13a, and broken off along the crack to form a fracture as shown in Figure 13b. Area A in Figure 13b is the cracked area formed during manufacturing, with a depth of about 2 mm, and length of about 9 mm. Areas B and C are the cracked extension areas formed by an external force.

**Figure 14:**
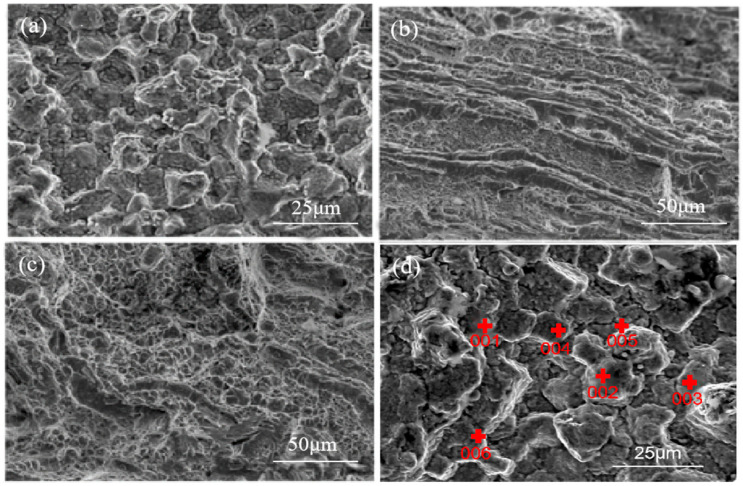
Morphology of the crack fracture in different zones. (**a**) zone A; (**b**) zone B; (**c**) zone C; (**d**) test points of zone A.

Figure 14 shows the crack fracture micro-morphology; it can be seen that the micro-morphology of A-zone is rough, and with a typical feature of brittle fracture along the grain, and grain boundary brittleness is the essential cause of the crack formation and extension [24,25,26]. Figure 14b shows that the micro-morphology of zone B has a rotten wood-like morphology, which belongs to the lamellar abnormal microstructure; Figure 14c shows that the micro-morphology of zone C is normal. The fracture extends from zone A to zone C.

Cracked area A of the micro-area elemental composition was analyzed by EDS; test points are shown in Figure 14d, and micro-area elemental composition results are shown in Table 5. It can be seen from Figure 14d, at the cracked areas marked 001, 003, and 006, that the Cr content is 1.53%, 1.44%, and 1.38% higher than the average content of 1.16%, and with abnormally high O content of 3.72%, 2.35%, and 2.76%, so it can be determined that Cr oxides existed at these places, according to the normal parts. At the areas marked 002, 004, and 005, Si content is 0.32%, 0.27%, and 0.25% higher than the average content of 0.18%, and with an abnormally higher O content of 2.51%, 2.12%, and 2.33%.

**Figure 15:**
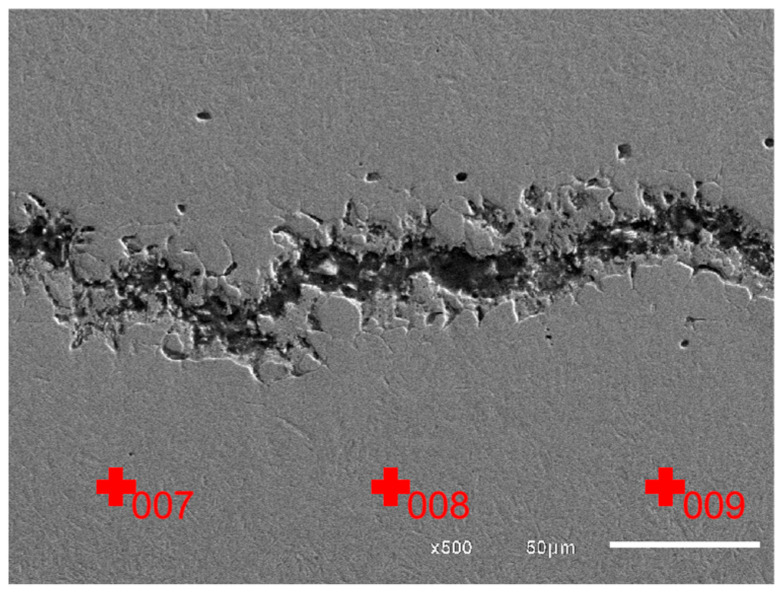
Test points away from the crack.

Figure 15 shows the EDS test points located away from the crack, and micro-area element content results are shown in Table 5. It can be seen that O content at the area away from the crack marked by 007, 008, and 009 is much lower than that at the crack area, being 0.35%, 0.48%, and 0.39%, respectively. Therefore, it can be determined that the higher O content in the crack area marked by 001 to 006 is not caused by oxidation after fracture.

Combining the results of Figure 14d and Figure 15 and Table 5, it can be determined that there exist oxides in the raw material, which corresponds to the white microstructures shown in Figure 8, Figure 9, Figure 10 and Figure 11. And based on the abnormally high content of Cr, Si, and O, it be inferred that the oxides which existed in the raw materials are most likely CrO_3_ and SiO_2_ [27], which belong to metallurgical defects existing in the raw materials.

## 4. Analysis and Discussion

It is well known that the possible cause of the cracks can be the defects in the raw materials, including the original cracks and oxide inclusions, improper quenching process parameters and untimely tempering, or improper grinding methods caused by excessive grinding stresses, and in this research, due to the fact that all the technical requirements of the products after heat treatment were met, including that surface hardness, CHD, and core hardness are qualified, and the hardness values at the same depth near the crack are very close to those away from the crack, it can be inferred that the crack is not caused by an improper heat treatment process. Meanwhile, the heat treatment limit test and residual stress detection confirmed that the heat treatment process is reasonable, and the stress formed during the carburizing and quenching process will not cause cracks as long as there are no existing defects in the raw materials.

And if there are cracks prior to quenching, the crack surface is supposed to result in decarburization due to the high carbon content after carburizing. While the microstructure shows that there is no typical decarburization characteristics on the crack surface of the parking ratchet, it can be concluded that the crack of the parking ratchet is not generated prior to heat treatment. And the characteristics of an almost linear crack and no grinding burn on the cracked surface can determine that the cracks are not generated during the grinding process, in other words, the cracks do not result from the improper grinding method with excessive grinding stresses.

Finally, from the crack fracture micro-morphology and EDS results of the micro-area elemental composition, it can be determined that there exist inclusions, including non-metallic oxides and metallic oxides in the raw materials, and it is well known that the inclusions have a strong tendency to cause cracks during quenching due to the big difference in crystal structure and severe mismatch in deformation with matrix, even though the quenching process is reasonable. Therefore, the root cause of cracking for the parking ratchets is attributed to the existence of oxide inclusions in the raw materials.

## 5. Conclusions

Cracking occurred in the grinding surface of the parking ratchet during quenching and grinding processes after carburizing; the goal of this research is to clarify the root cause of the generation of the cracks, after systematical analysis, and the main conclusions can be drawn as follows:

(1) The heat treatment process is reasonable and the crack is not caused by an improper heat treatment process, since all the technical requirements of the parking ratchets after heat treatment were met, and the hardness values at the same depth near the crack are very close to those away from the crack. The heat treatment limit test further confirmed that the heat treatment process is reasonable, since no cracks occur for the parking ratchets by using the current heat treatment process as long as no defects existed in the raw materials.
(2) The crack of the parking ratchet is not generated prior to heat treatment, since the microstructure shows no typical decarburization characteristics on the crack surface of the parking ratchet.
(3) The grinding process is reasonable, since higher magnification morphology of the surface crack shows no grinding burn on the crack surface, and the grinding depth, grinding pressure, and grinding speed are all within the control range.
(4) The root cracking cause of the parking ratchets is the existence of the oxide inclusions in the raw materials, which have a strong tendency to result in cracks during quenching due to the big difference in crystal structure and severe mismatch in deformation with matrix. Therefore, in order to avoid this kind of crack, it is necessary to thoroughly check the raw material of the parking ratchet by a magnetic particle detector prior to manufacturing and make sure qualified raw materials are used.

## Figures and Tables

**Table 1:** The chemical composition of 20MnCrS5 HL (wt %).

Element	C	Si	Mn	P	S	Cr	Fe
Technical value	0.17–0.22	≤0.40	1.10–1.30	≤0.025	0.02–0.04	1.0–1.3	Bal.
Measured value	0.200	0.18	1.158	0.016	0.028	1.164	Bal.

**Table 2:** The results of hardness and CHD.

Test Item	Surface Hardness (HV1)	Core Hardness (HV1)	CHD (mm)
Technical requirements	≥674	345–402	0.7–1.0
Measured values	706.2	393	0.76

**Table 3:** The results of limit test for carburizing and quenching.

Clusters	Clusters 1	Clusters 2
Number of tests (pieces)	5	5
Number of cracks (pieces)	0	0

**Table 4:** Surface and subsurface residual stresses.

Depth (μm)	Normal Part (MPa)	Cracked Part (MPa)
0	−903	−858
500	−325	−287

**Table 5:** Micro-area elemental composition results of fracture zone A (wt %).

Position	O	Si	Cr	Fe	Total
001	3.72	0.18	1.53	94.57	100
002	2.51	0.32	1.05	96.12	100
003	2.35	0.19	1.44	96.02	100
004	2.12	0.27	1.07	96.54	100
005	2.33	0.25	1.05	96.37	100
006	2.76	0.19	1.38	95.67	100
007	0.35	0.14	1.15	98.36	100
008	0.48	0.17	1.11	98.14	100
009	0.39	0.17	1.10	98.34	100

## Data Availability

The original contributions presented in this study are included in the article. Further inquiries can be directed to the corresponding author.
